# Examining the Impact of Reading Fluency on Lexical Decision Results in French 6th Graders

**DOI:** 10.1162/opmi_a_00140

**Published:** 2024-05-05

**Authors:** Marie Lubineau, Cassandra Potier Watkins, Hervé Glasel, Stanislas Dehaene

**Affiliations:** Cognitive Neuroimaging Unit, CEA, INSERM, Université Paris-Saclay, NeuroSpin center, 91191 Gif/Yvette, France; Collège de France, Université Paris-Sciences-Lettres (PSL), 11 Place Marcelin Berthelot, 75005 Paris, France; Reference centre for the neuropsychological evaluation of children (CERENE), Paris, France

**Keywords:** lexical decision, reading, middle school, fluency

## Abstract

Purpose: How does lexical decision behavior vary in students with the same grade level (all students were in their first year of middle-school), but different levels of reading fluency? Here, we tested a prediction of the dual-route model: as fluency increases, variations in the results may reflect a decreasing reliance on decoding and an increasing reliance on the lexical route. Method: 1,501 French 6^th^ graders passed a one-minute speeded reading-aloud task evaluating fluency, and a ten-minute computerized lexical decision task evaluating the impact of lexicality, length, word frequency and pseudoword type. Results: As predicted, the word length effect varied dramatically with reading fluency, with the least fluent students showing a length effect even for frequent words. The frequency effect also varied, but solely in proportion to overall reading speed, suggesting that frequency affects the decision stage similarly in all readers, while length disproportionately impacts poor readers. Response times and errors were also affected by pseudoword type (e.g., letter substitutions or transpositions), but these effects showed minimal variation with fluency. Overall, lexical decision variables were excellent predictors of reading fluency (r = 0.62). Conclusion: Our results highlight the variability in middle-school reading ability and describe how a simple lexical decision task can be used to assess students’ mental lexicon (vocabulary) and the automatization of reading skills.

## INTRODUCTION

Lexical Decision (LD) is a classic psycholinguistics paradigm requiring participants to classify visually presented stimuli as words or pseudowords. It has been widely used in cognitive science and provides well-replicated measures of key components of the visual word recognition process, which are captured by the Dual Route Model of reading (Coltheart et al., 2001; Fiebach et al., 2002). This model describes the reading process as the outcome of two competing pathways. On the one hand, the lexical pathway lends itself to fast fluent reading of known words stored in the mental lexicon. This lexicon is affected by word frequency, such that most frequent words are quicker to retrieve than less frequent ones. On the other hand, the sublexical pathway enables reading unknown words or pseudowords, via the application of grapheme-phoneme correspondence rules. The necessity to decode in order to access sound and thereby meaning (or rejection of meaning) causes response time (RT) to stimuli processed via the sublexical pathway to be strongly affected by the number of letters in the word (Acha & Perea, 2008; Bijeljac-Babic et al., 2004; Di Filippo et al., 2006; Martens & de Jong, 2006).

With LD, it is very easy to measure the length, lexicality and frequency effects that arise from the use of one of these two routes by varying the lexicality of stimuli (word or pseudoword), their length in letters, and their frequency (for words only). Numerous cross-sectional studies in alphabetic languages, mentioned hereafter, have investigated how these effects change with reading acquisition. For example, children, compared to adults, are affected by a more pronounced word length effect, as they rely more heavily on sublexical procedures when learning to read (Acha & Perea, 2008). When reading becomes efficient, the word length effect fades for words ∼3–8 letters long, indicating that the lexical pathway has become fully operational and that reading has become automatic (Ferrand et al., 2010; New et al., 2006; Weekes, 1997). This decrease arises between 2^nd^ and 5^th^ grade, indicating a slow transition from serial grapheme–phoneme mapping to a greater reliance on lexical knowledge, with differences between languages. In transparent languages, such as Italian, the length effect already vanished in 3^rd^ grade (Zoccolotti et al., 2005, 2009), whereas in more opaque languages such as French, length effects are still significant in 5^th^ graders (Bijeljac-Babic et al., 2004).

The decrease in the length effect is concomitant with the appearance of a frequency effect (Faust et al., 1999). Indeed, as word reading time and accuracy cease to be influenced by length, they become increasingly affected by frequency (Brysbaert et al., 2011; Burani et al., 2002; Grainger & Segui, 1990; Ratcliff et al., 2004). At this stage, reading is characterized by a linear relationship between the logarithm of frequency and RT (Norris, 2006). This transition towards faster lexical procedures can take from one to two years, depending on the transparency of the orthography in a given language, with a faster transition for more transparent languages (Schmalz et al., 2013). In this study, we focus on the French language, which has an opaque orthography characterized by numerous graphemes with diverse phonemic realizations. It is noteworthy that the frequency effect in reading acquisition typically appears after nearly a year of formal reading instruction (Sprenger-Charolles et al., 1998). Additionally, the lexicality effect generally emerges for high-frequency words around the 3^rd^ grade (Araújo et al., 2014; Di Filippo et al., 2006; Juphard et al., 2004; Sela et al., 2014).

The results of the studies mentioned above however, do not tell us anything about how students at the same grade level differ, although it is well known that large differences in reading literacy exist within grade. This is particularly true at the start of middle-school in France (6^th^ grade), where this study took place, where many students enter without the skills needed for independent reading (Andreu et al., 2021, 2022). To our knowledge, the only current studies that looked at students in the same grade compared dyslexics and non-dyslexics (Castles, 2006; Martens & de Jong, 2006; Zoccolotti et al., 2005). Some studies also looked at the impact of reading level on lexical decision results, such as Yeatman et al. who looked at variations in the length effect with reading level in a wide range of ages (Yeatman et al., 2021). However, the question of how lexical decision behavior is impacted by reading level has never been explored in children within the same grade, maybe because of the low number of participants per grade in many studies (Zoccolotti et al., 2005 included 30 participants per grade; Martens & de Jong, 2006 included 22 participants per grade; Araújo et al., 2014 included 19 participants in three different grades). Here, using our large-scale study conducted on a representative sample of the French 6th graders, we aimed to evaluate the hypothesis that the results observed in cross-sectional studies can be replicated among students within the same grade. We predicted that the same diversity of LD behaviors, reflecting the differential use of the two reading routes as a function of reading expertise, could be observed within the same age and grade level, as previously observed cross-sectionally. Such a result would be in line with neuroimaging findings showing that it is reading fluency, rather than grade or age, that determines the development of reading circuits (Dehaene et al., 2010; Dehaene-Lambertz et al., 2018; Feng et al., 2022; Monzalvo et al., 2012).

### How Do Lexicality, Length and Frequency Effects Vary Within a Grade?

Our first aim was to characterize the effects of lexicality, length, and frequency as a function of reading level in students with the same grade level. We do so on the usual measures of response time and error rates but also on normalized RTs (*z* scores), obtained by subtracting the subject’s mean and dividing by the subject’s standard deviation (Faust et al., 1999). Given our anticipation of slower performance in the lexical decision (LD) task among participants with poorer reading proficiency, this measure allows to examine whether slowness alone can account for the larger observed effects on response times (RTs) among less fluent individuals. Indeed, slower participants often exhibit more pronounced RT effects compared to faster participants (Faust et al., 1999). If those effects occur at the decision stage, which is thought to involve a stochastic accumulation of evidence, then one would predict effect size to be proportional to the standard deviation of RTs across trials (Ratcliff & Rouder, 1998; Sigman & Dehaene, 2005). Sigman & Dehaene show how response times can be decomposed into a series of perceptual, decision and motor stages, with the non-decision showing little variability compared to the decision itself in participants’ response times. If all participants used the same decision procedure, and varied only in the strength of the evidence and/or the threshold for decision making, then the length and frequency effects observed on response times should disappear once response times are normalized into *z*-scores. If those effects remain, it means that larger procedural differences exist between groups, over and above a mere difference in the speed of decision making.

### How Are the Responses to Pseudowords Affected by Reading Level?

Another goal of our work was to better understand how students with different levels of reading ability process pseudowords. Our aim here was to map the proportions of different types of errors, and their potential modulations with reading level. From a practical point of view, this will make it possible to determine which errors are still present in very good readers and which errors disappear as a reader becomes fluent. To this aim, we designed different types of word-derived pseudowords, also called “traps” because they must be rejected in spite of their often close similarity to words.

Much prior research has demonstrated an effect of the orthographic similarity of pseudowords to words, showing that the more similar a pseudoword is to a word, the more difficult it is to reject it (Davis & Bowers, 2006; Ferrand & Grainger, 1993; Grainger & Segui, 1990). Responses to pseudowords are longer for pseudowords that are very similar to words, than for pseudowords that are less similar to words (Andrews, 1989; Coltheart et al., 1977). This similarity is classically measured using Coltheart’s N (Coltheart et al., 1977), which considers as “neighbors” two strings of the same length that differ by only one letter. Other measures, such as the orthographic Levenstein Distance 20 (OLD20), compute the distance between two words as the minimum number of operations required to move from one word to another using operations of substitution, transposition, addition, or deletion, and then average this distance for the 20 closest words to the target word (Yarkoni et al., 2008). Here, we explored a wide range of pseudoword types, based on the presence of transposed or mirror letters, as well as misspelled words.

Both developing and skilled readers can make errors characterized by internal letter transpositions (i.e., reading “from” as “form”) (Friedmann & Gvion, 2001; Paterson et al., 2015). In LD tasks, children show a higher tendency to misclassify pseudowords with transposed letters. This effect initially intensifies with reading acquisition and subsequently diminishes to its minimum level among skilled readers. (Grainger et al., 2012). We expected our participants to be slower and less accurate when processing letter-transposition pseudowords than their double-substitution controls, an effect that would be the highest for our most fluent readers.

Regarding mirror generalization, it is an early predisposition of the pre-reader’s brain that must be inhibited or superseded when learning to read (Dehaene, 2009; Dehaene et al., 2015; Kolinsky et al., 2011; Pegado et al., 2011, 2014). Indeed, identifying that two mirror letters are different is harder than differentiating between two non-mirror letters (Ahr et al., 2016). In addition, the sounds /p/-/b/ and /b/-/d/ are very close phonologically. Thus, we expected that processing pseudowords containing mirror substitutions, such as ‘dateau’ instead of the French word ‘bateau’, would require a greater effort than processing pseudowords arising from an equivalent, non-mirror letter substitution (e.g., ‘fateau’; English equivalents would be ‘dalance’ [derived from ‘balance’] versus ‘falance’).

As students with reading deficits often confuse letter-sound rules (Rack et al., 1992), we finally introduced misspelled words that would sound like a word only if a wrong, shallower grapheme-phoneme correspondence rule was applied (e.g. “bage”, which could be read as the word “bague” if the reader did not know that in French, the letter “g”, when followed by an “e”, should be read as “j”). Strictly speaking, these pseudowords are not pseudohomophones, but they share some properties with them, including a spelling close to a real word and the fact that they could be pronounced as a word (by readers who do not master all of the French contextual rules). Prior research using lexical decision showed that students exhibit major difficulties distinguishing between words and pseudohomophones (Bergmann & Wimmer, 2008), and that those errors decrease in the course of reading development (Grainger et al., 2012). Thus, we expected higher RTs and error rates for orthographic traps than for control word approximations, and a reduction of this effect as fluency increased.

Errors on orthographic traps may be an outcome of poor reading experience exasperated by difficulties in learning French, a language with an opaque orthography. We hypothesized that all readers would be slower to classify pseudowords based on their distance to a real word, but that poor readers would be further penalized by orthographic traps.

### Can Lexical Decision Results Predict Fluency Scores?

Finally, our last goal was to investigate the predictive value of the LD task on fluency. LD was shown to be highly correlated with a standardized fluency measure of oral reading for children and adults (Gijsel et al., 2004; van Bon et al., 2004; Yeatman et al., 2021). Here, we investigated how LD accuracy and RT varied in relation to text reading fluency. By correlating these tasks, we aimed to further our understanding of how elementary measures of single-word phonological and orthographic processing, acquired during LD, relate to fluent text processing.

## METHODS

### Participants

Within the framework of the French national evaluations taken by all 6th-grade students, which marks the commencement of middle school, we implemented our LD task in a student panel. These evaluations on expected grade-level French and Math abilities are done individually on computer, with the exception of a one-minute oral reading fluency test. The administration and data collection processes were overseen by the Direction of Evaluation, Prospective, and Performance (DEPP), the ministerial service responsible for education statistics in France (website: www.education.gouv.fr/direction-de-l-evaluation-de-la-prospective-et-de-la-performance-depp-12389).

The panel taking the LD task consisted of 3,472 students chosen by the DEPP as representative of French population. The DEPP was also able to provide us with the reading fluency scores for 2,194 of these students. To examine the impact of reading ability on lexical decision results, we only kept students who had completed both tests. No differences in gender or socio-economic status between our initial and reduced sample were found. Out of these 2,194 students, only 1,501 completed the LD task entirely. We found that the lower the student’s scores in fluency, the more they abandoned the lexical decision along the way. Their partial data were still collected, but the number of trials performed was too low to be analysed. Thus, the results described in this article concern the 1,501 students (806 girls and 695 boys, mean age = 11.0 y.o.) who had a complete LD dataset (120 trials) and reading fluency scores. This sample was still representative of the French population in terms of socio-economic status, as the IPS (“indice de position sociale, i.e., socio-economic status [SES]), an indicator determined by the Ministry of Education based on the occupation of the student’s parents (Rocher, 2016), did not differ between the original sample designed by the DEPP and our subsample (*t*(2873.3) = −0.41, *p* = 0.68).

### Reading Fluency Test

Within the National Evaluations, student fluency was assessed by the standarized text, Le Géant égoïste included in the BALE battery (Jacquier-Roux et al., 2010). This text consists of 206 words, spread over 15 lines. The teacher administered this portion of the national evaluation individually. In a quiet room, the student was instructed to read the text aloud for one minute, as accurately as possible in normal reading speed. Teachers reported the student’s number of words correctly read in one minute. During the test, teachers were asked to time the student and identify incorrectly read words. When the student hesitated or repeated himself/herself, but ended up reading the word correctly, the trial was considered correct. At the end of the test, teachers reported the student’s number of words correctly read in one minute. This measure constitutes the fluency score.

### Lexical Decision Task

The LD task was included in the computerized portion of the national assessment. Students worked individually with headphones in a group setting in the school’s computer lab. The task started with written and oral instructions: “For this exercise, decide as fast as possible if what is written on the screen is a real word or a trap. Press M for a word and Q for a trap.” This was followed by a video demonstration for each item category (word or trap). The position of the response keys was not counterbalanced across participants. Students clicked a button when they were ready to start. Each item remained in the middle of the screen until the student responded by pressing ‘Q’ or ‘M’ or was skipped after a 5000ms time limit. Audio-visual feedback was provided. Positive audio feedback increased in tone with consecutive correct responses to encourage pursuit of winnings streaks. We collected measures of accuracy and response time (RT in ms). There were twelve different lexical decision modules, each composed of 120 stimuli, 60 words and 60 pseudowords (see below). Students were randomly assigned to a module. The whole task was administered in one block, and stimulus order was randomized within each student for a total duration of less than 10 minutes.

#### Word Stimuli.

We first extracted all mono-lemmatic and mono-morphemic words from the Lexique 3.83 database (New et al., 2004) with a length of four to eight letters and a frequency higher than three per million. We manually excluded all potentially offending, inappropriate or foreign words, thus resulting in a stimulus set of 3,656 words. These items were then separated into four different frequency bands: very frequent, frequent, rare, and very rare (see Table 1 for details). 12 modules were designed using this database. For each module, we randomly selected 3 words from each frequency category and each length, resulting in a factorial design with length (5 levels, 4–8 letters) and frequency (4 levels) as factors, for a total of 60 words per module.

**Table 1.** Characteristics of words stimuli and examples.

Frequency category	Length				
	4 letters	5 letters	6 letters	7 letters	8 letters
Very frequent (greater than 100 per million)	beau (nice)	avion (plane)	visage (face)	message (message)	déranger (disturb)
Frequent (40 to 100 per million)	acte (act)	usine (factory)	camion (truck)	étudier (study)	vaisseau (vessel)
Rare (10 to 40 per million)	vélo (bike)	alibi (alibi)	carnet (booklet)	complot (conspiracy)	éprouver (experience)
Very rare (3 to 10 per million)	cerf (deer)	maçon (bricklayer)	abolir (abolish)	stocker (store)	élégance (elegance)

#### Pseudoword Stimuli.

Using only words from the “very frequent” category, we built six categories of pseudowords *“traps”*, for a total pool of 1,196 items. For each module, we randomly selected two pseudowords for each type and each length, thus resulting in a factorial design with pseudoword category (6 levels, see below) and length (5 levels, 4–8 letters) as factors, and 60 pseudowords per module. We describe each of the pseudoword categories below. Examples are presented in Table 2.

**Table 2.** Characteristics of pseudowords stimuli and examples.

Pseudoword traps	Length				
	4 letters	5 letters	6 letters	7 letters	8 letters
Orthographic traps	bage (bague = ring)	plase (place = place)	inciet (inquiet = worried)	ésaiyer (essayer = try)	difisile (difficile = hard)
Word approximations	atio	ouvoi	jamure	répoure	voicider
Transpositions	ceil (ciel = sky)	juene (jeune = young)	geurre (guerre = war)	pafrois (parfois = sometimes)	regadrer (regarder = look)
Double substitutions	cuol (ciel = sky)	jaine (jeune = young)	gairre (guerre = war)	pansois (parfois = sometimes)	reganger (regarder = look)
Mirror substitutions	aibe (aide = help)	qièce (pièce = room)	dateau (bateau = boat)	musipue (musique = music)	qrochain (prochain = next)
Single substitutions	aite (aide = help)	gièce (pièce = room)	fateau (bateau = boat)	musijue (musique = music)	grochain (prochain = next)

##### Orthographic Traps.

These were misspelled words that could, by an erroneous grapheme-phoneme correspondence, sound like real word. These pseudowords were manually built by selecting all words in our pool with a given rule-based grapheme-phoneme correspondence, then over-regularizing it. We focused on the letters s, c, and g, whose pronunciation varies with context in French. The letter ‘s’ most frequently sounds as /s/ (e.g., in “sale”) except when a single ‘s’ is sandwiched between vowels (e.g., in “base”), causing it to sound /z/. Similarly, letters ‘c’ and ‘g’ respectively sound as /k/ and /g/ when followed by the vowels ‘a’, ‘o’ and ‘u’, but as /s/ and /Z/ when followed by the letters ‘e’ and ‘i’. As a result of those rules, the pseudoword ‘ausi’ should be read /ozi/, and the pseudoword ‘bage’ should be read /baZ/. A reader for whom these rules have not been consolidated might read these pseudowords as the words /osi/ (also) and /bag/ (ring).

##### Word Approximations.

Those were pseudowords entirely made of frequent French trigrams. To build them, we calculated the frequencies of all legal letter trigrams in our word pool. Only trigrams with frequency greater than 1/10,000 were retained. Pseudowords of 4 to 8 letters were then built solely from those frequent trigrams. We implemented a Markov process that (1) draws an initial trigram at random, in proportion to its frequency; (2) uses the last two letters to continue with the next trigram, again drawing randomly based on frequency, and so on. For example, the 4-letter pseudoword “arie” could be built using the frequent trigrams ‘ari’ and ‘rie’. Actual words were excluded by software and human inspection.

##### Letter Transposition.

Pseudowords of this category were constructed by inverting two adjacent internal letters of a word. The transposed bigram was composed of either two vowels or two consonants. Only pseudoword items whose bigrams exceeded a frequency of 1/10,0000 were kept. Bigram frequency was calculated using the same method described above for trigrams.

##### Double Substitutions.

This category, a control for transpositions, was built by substituting the same bigram in each transposed pseudoword with another random bigram with frequency higher than 1/10,000. Consonants were replaced by consonants, and vowels by vowels. All the resulting bigrams from this substitution were controlled to ensure that their frequency exceeds a threshold of 1/10,000.

##### Mirror Substitutions (Mirroring of Letters b d p q).

This category was generated by mirroring mirror letters in the following way: p → q ; q → p ; b → d ; d → b. Items were only kept if the transformation yielded a pseudoword.

##### Single Substitutions (Substitution of Letters b d p q).

In this category, a control for the mirror substitution traps, letters b d p q were substituted with a non-mirror letter: p → g ; q → j ; b → f ; d → t.

### Data Analyses

We used the fluency score of the student as a measure of reading automatization. Since our twelve lexical decision modules, by design, did not differ significantly in terms of length, OLD20 (*F*(11, 1427) = 0.24, *p* = 0.99), letter frequency (*F*(11, 1427) = 0.68, *p* = 0.76), bigram frequency (*F*(11, 1427) = 0.27, *p* = 0.99) and word frequency (*F*(11, 708) = 0.97, *p* = 0.47), we decided to analyze all the data together, rather than module by module. Response time (RT) on correct answers exceeding 200ms were included in a linear mixed-effect model, incorporating fluency level (quintiles 1–5), lexicality (word, pseudoword), length (4–8 letters), and word frequency (4 levels ranging from very frequent to very rare) as fixed effects. Subject and stimulus were treated as random effects. We decided to conduct our analysis with the fluency quintiles as a 5-level factorial variable, rather than a single continuous variable, because the variations in length and frequency effects as a function of fluency were clearly non-linear (the increase in the slopes of some of these effects between groups did not follow a straight line, but became disproportionately slower for the less fluent groups). Similar results were obtained when running our analysis with fluency as a continuous variable, and detailed statistics are reported in Tables S1 and S2 in supplementary materials.

To compute our mixed effect models, we used the *mixed* function from R’s *afex* package with the following formula:

$$\text{dv}∼X_{1}*X_{2}*\ldots *X_{n}+\left(1|\text{subject}\right)+\left(1|\text{stimulus}\right)$$

*X*_*i*_ represents the combination of our interacting fixed effects. *dv* is either accuracy of the answer or RT. For RT, we computed a classical mixed effect model (Baayen et al., 2008) while for accuracy we used a logistic mixed effect regression with the binomial link function (Jaeger, 2008). Significance was computed using the Satterthwaite’s degrees of freedom for RT and the Likelihood ratio test (LRT) for accuracy. All analyses used a significance threshold of *α* = 0.05.

Follow-up analyses were done using the simple effect analysis where we split the data into subsets according to the modulating variable(s) and recomputed the model with only the remaining variable(s).

In addition to the effects, we also reported conditional and marginal R^2^, calculated using R *performance* package, as a measure of the variance explained by our fixed and random variables (Nakagawa et al., 2017).

To check if the slowness of less fluent readers alone could explain why they showed larger (absolute) effects on RTs, we turned our raw RT into *z*-scores by subtracting from each subject’s RT their overall mean and then dividing by their overall standard deviation (Zoccolotti et al., 2008), after verifying that our data fitted the conditions to apply Faust et al.’s (1999) rate and amount model (RAM).

Finally, to investigate the predictive value of the LD on reading fluency, we used both simple and multiple linear regression. The predictive variables were the student’s median RT on correct answers across all trials, global error rate, slope of the length effect, slope of the frequency effect on words, efficiency score (ratio of accuracy by mean RT). The slopes of the length and frequency effects were calculated for each student by taking their median RT on correct answers for each length (resp. for each frequency category), and measuring the coefficient of a linear model fitted to these data. In our multiple linear regression model, variables were all normalized to facilitate results interpretation.

## RESULTS

**Figure 1.**
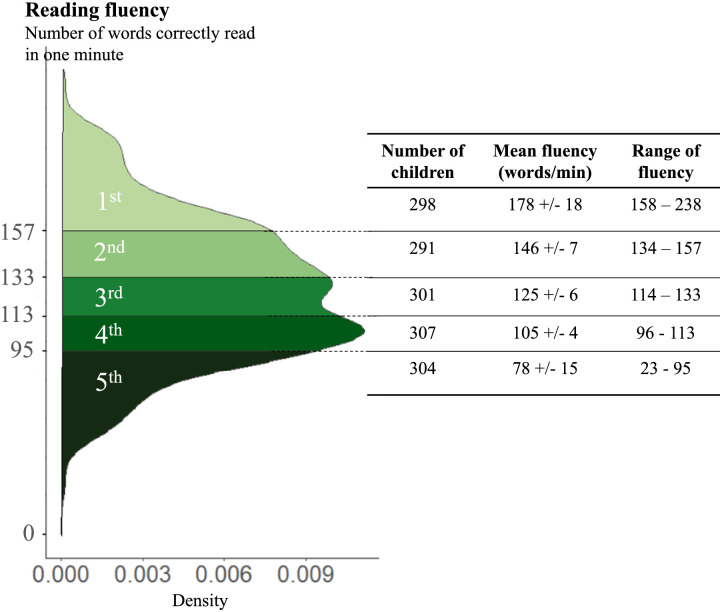
Distribution of participant’s reading fluency on the National Evaluation. Reading fluency was assessed by measuring the number of words correctly read in a text, in one minute. Color graduation from dark to light corresponds to fluency group levels, with light green corresponding to the most fluent students. Each quintile group represents 20% of the tested population.

Figure 1 shows the distribution of text reading fluency in our sample, i.e. the number of words correctly read in one minute. Since the primary goal of our project was to assess within-grade variability in the size of the lexicality, length and frequency effects in the LD task results, we first separated students into five groups based on their fluency score, each quintile group containing 20% of the students. Fluency quintile 1 refers to the best readers and 5 to the poorest readers.

These quintiles are indicative of the expected Gaussian reading levels of pupils for this grade level according to the test’s standardized norms. At the start of 6^th^ grade students are expected to be capable of correctly reading 127.16 words per minute in this text, with a standard deviation of 29.41. Within our sample, 47.7% of the students perform above this norm, while 57.8% fall within the range of plus or minus one standard deviation. Moreover, 5.06% of the pupils exhibit reading abilities below two standard deviations.

### Length and Lexicality Effects

**Figure 2.**
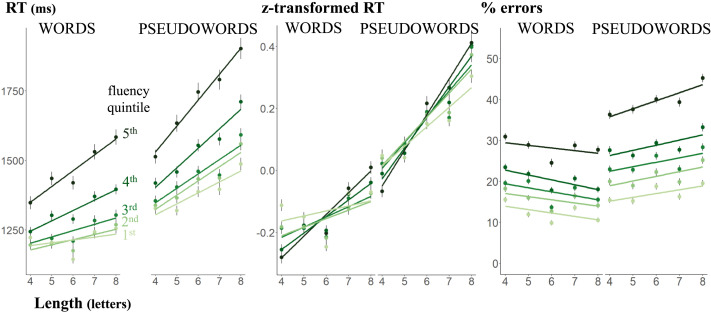
Length and lexicality effects on response times on correct answers (RT), Z-transformed RTs, and error rates. Each point represents the mean RT or error rate as function of word length and fluency. Error bars represent one standard error of the mean. The slopes are the linear regression associated with the points.

LD results are plotted in Figure 2 as a function of lexical status and length. In agreement with the literature on LD RT, responses to words were faster than to pseudowords, *F*(1, 1063.1) = 213.20, *p* < .0001; better readers read faster, *F*(4, 1478.9) = 58.86, *p* < .0001; RT increased with word length *F*(1, 1062.5) = 73.85, *p* < .0001. All three two-way interactions were significant: fluency × lexicality, *F*(4, 135933.4) = 16.55, *p* < .0001; fluency × length, *F*(4, 135826.6) = 50.37, *p* < .0001; lexicality × length, *F*(1, 1062.6) = 7.63, *p* = 0.006. There was, however, no significant three-way interaction between fluency, lexicality, and length *F*(4, 135833.3) = 0.23, *p* = 0.92. We observed a marginal r^2^ of 0.049 and a conditional r^2^ of 0.288.

Performing simple effect analysis on the significant interaction between length and fluency, we found that that all quintiles exhibit a significant length effect. The steepness of the slope of this effect increased, as fluency level decreased. Looking at the interaction between length and lexicality, we found that both words and pseudowords exhibit a significant length effect, which slope was steeper for pseudowords than for words. Surprisingly, we observed a clear dip in RT for 6-letter words across all frequency quintiles, probably due to the specific items selected in this category.

Since the neighborhood density of both words and pseudowords, quantified using OLD20 (Yarkoni et al., 2008), was significantly correlated with item length in every category of words and pseudowords (Figure S1), we also ran a model with OLD20 as an additional covariate. Our results remained unchanged. This conclusion also applies to the models on error rate as well as the models described in the paragraph on the interaction between frequency and length.

As expected, less fluent readers were also slower in the lexical decision task. Could a slower decision process alone explain why less fluent subjects showed a larger (absolute) effect of word length on RTs? To answer this question, we turned our raw RT into *z*-scores. The results remain unchanged. Our mixed effect model on z-transformed RTs showed that all effects were significant except the three-way interaction, *F*(4, 137202.4) = 0.45, *p* = 0.77. Crucially, the fluency by length interaction remained significant, *F*(4, 137202.4) = 30.46, *p* < .0001, indicating that the reduction of the length effect as fluency increased went beyond what could be due solely to faster responses. Fluent readers are not just faster, but have a genuinely smaller length effect.

We next turned our attention to comparing error rates. Results mirrored those for RT, with a marginal r^2^ of 0.058 and a conditional r^2^ of 0.278. All subjects were more accurate for words than pseudowords, *χ*^2^(1) = 91.85, *p* < .001, and better readers were more accurate than their counterparts, *χ*^2^(4) = 595.89, *p* < .001. The main effect of length was not significant, *χ*^2^(1) = 0.01, *p* = 0.92, but a significant interaction was found between length and lexicality, *χ*^2^(1) = 14.16, *p* < .001: greater length increased the likelihood of errors for pseudowords, but decreased it for words. We also found a significant interaction between fluency and lexicality, *χ*^2^(4) = 21.02, *p* < .001, which highlights the increasing difference in performance between words and pseudowords as the level of fluency decreases. There were no interaction between fluency and length, *χ*^2^(4) = 9.36, *p* = 0.053, nor a three-way interaction, *χ*^2^(4) = 1.00, *p* = 0.91. In the absence of an interaction between fluency and length, we conclude that across all trials, none of the quintiles exhibits any significant length effect.

Considering the educational implications of the present work, we also ran the models described above with IPS (equivalent of SES) as a covariate. Our results remained unchanged. This conclusion also applies to all the models described in the rest of this article.

### Frequency Effect on Words

**Figure 3.**
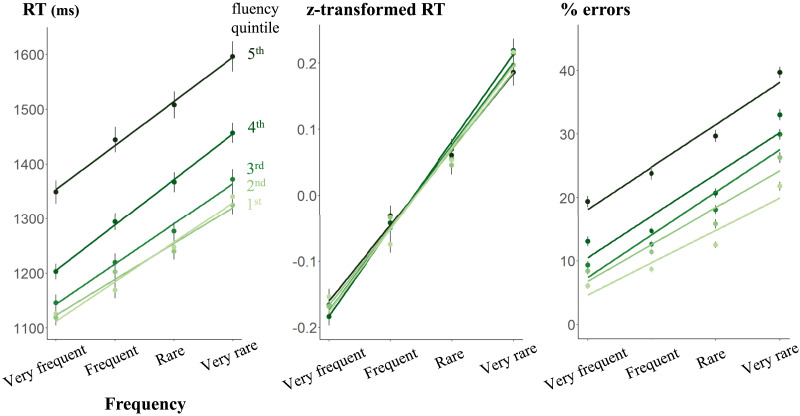
Frequency effects on response times on correct answers (RT), Z-transformed RTs, and error rates. Each point represents the mean RT or error rate as a function of frequency and fluency. Error bars represent the standard error of the mean. The slopes are the linear regression associated with the points.

Subsequently, we focused our investigation on word items within the LD task, specifically examining the influence of word frequency and its relationship with fluency variations. Correct RTs and error rates sorted by the four bands of frequency appear in Figure 3. Both RT and accuracy were affected by a significant main effect of frequency (RT: *F*(1, 561.66) = 101.40, *p* < .0001; accuracy: *χ*^2^(1) = 219.05, *p* < .001). Again, there was a main effect of fluency quintile (RT: *F*(4, 1461.01) = 54.23, *p* < .0001; accuracy: *χ*^2^(4) = 422.22, *p* < .001) and a significant interaction between fluency and frequency (RT: *F*(4, 70582.79) = 4.59, *p* = 0.001; accuracy: *χ*^2^(4) = 30.93, *p* < .001) due to the fact that the slope of the frequency effect decreased in the most fluent readers. Analyses restricted to each fluency quintile showed that, for both RT and accuracy, performance for all fluency quintiles dropped with less frequent words. The slope of decreased performance was only less steep for our best performers. We observed a marginal r^2^ of 0.035 on RT and 0.095 on error rate, and a conditional r^2^ of 0.250 on RT and 0.287 on error rate.

Again, we used z-transformed RTs to examine whether the reduction in the frequency effect as fluency increased was solely due to faster overall responses. The main effect on frequency remained significant, *F*(1, 620.63) = 116.36, *p* < .0001, but the two-way interaction vanished, *F*(4, 71977.60) = 1.06, *p* = 0.37. This finding suggests that, once the speed of their responses was considered, the frequency effect on RT was actually identical for all students, no matter their fluency level.

### Interaction Between Length and Frequency

**Figure 4.**
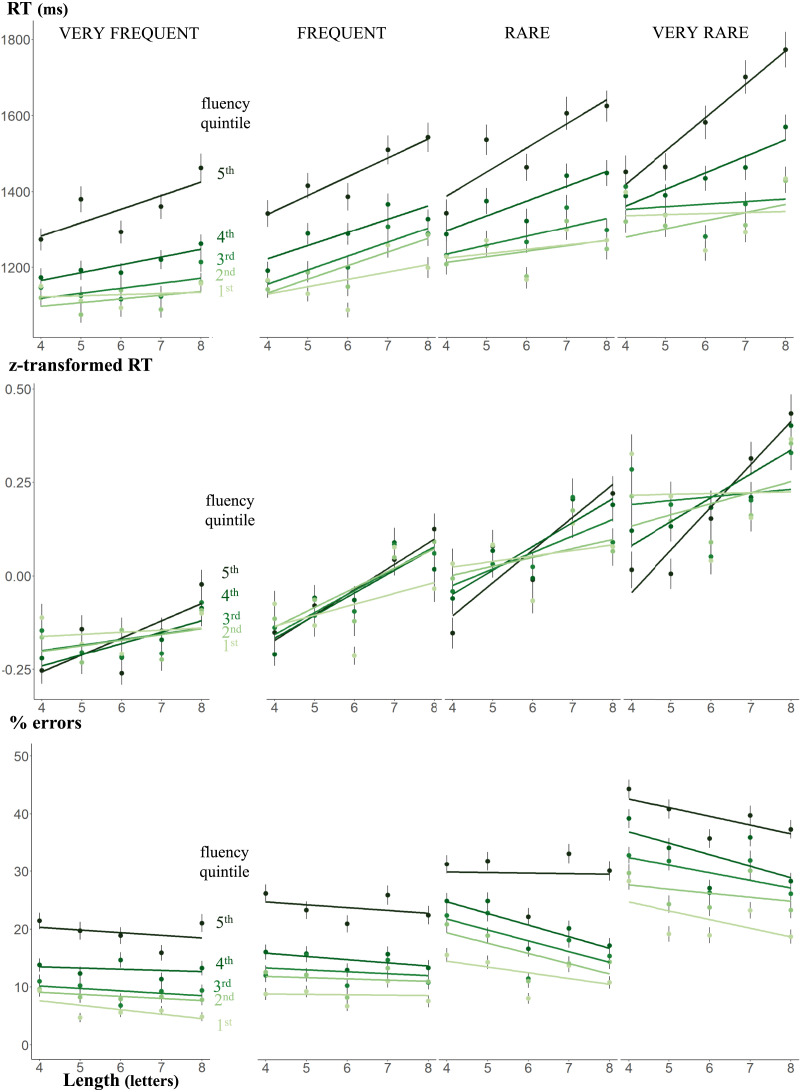
Interaction between length, frequency and fluency on response times on correct answers (RT), Z-transformed RTs and error rates. Each point represents the mean RT or error rate as a function of length, frequency, and fluency. Error bars represent the standard error of the mean. The slopes are the linear regression associated with the points.

Our large sample also allowed us to investigate the interaction between length, frequency, and fluency (Figure 4). Our prediction was that these variables should have a 3-way interaction on RTs because (1) fluent readers would show little or no length effect, regardless of frequency, as they consistently rely on the fast lexical route; (2) less fluent readers would show an increasingly marked length effect as word frequency decreases, because lower frequency decreases the probability that they use the lexical route.

In a general linear model restricted to words only (RT: marginal r^2^: 0.041, conditional r^2^: 0.253; accuracy: marginal r^2^: 0.098, conditional r^2^: 0.288), the predicted three-way interaction between fluency quintile, word frequency and length was significant for RT, though not for accuracy (RT: *F*(4, 70564.14) = 5.59, *p* = 0.0002; accuracy: *χ*^2^(4) = 2.76, *p* = 0.60). Figure 4 depicts the basis of this triple interaction: RTs were affected by the frequency × length interaction, and as predicted, this interaction decreased as fluency increased. A simple effect analysis on length, separately for each fluency × frequency level, showed a clear trend: the most fluent students exhibited no length effect, no matter the frequency of the words, whereas the least fluent student showed a significant length effect even for very frequent words.

Results on z-transformed RTs were almost exactly the same as for RT, except that, as expected, there was no longer a significant main effect of fluency quintile, *F*(4, 71970.80) = 1.30, *p* = 0.27, as well as any significant interaction between word frequency and fluency, *F*(4, 71979.29) = 0.99, *p* = 0.41, as previously reported. The interaction between length and frequency was also not significant, *F*(1, 598.16) = 0.95, *p* = 0.33. As the three-way interaction between length, word frequency and fluency level remained significant, our conclusions remained unchanged.

### Processing of Pseudowords

**Figure 5.**
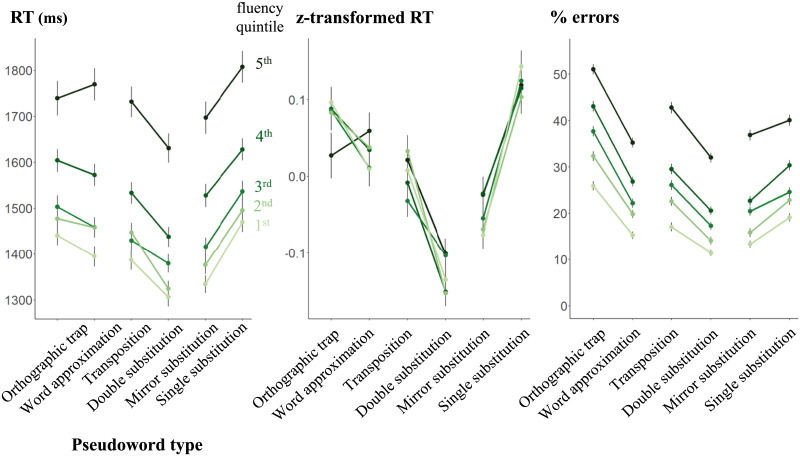
Responses to pseudowords: response times on correct answers (RT), Z-transformed RTs, and error rates. Each point represents the mean RT or error rate as a function of pseudoword type and fluency. Error bars represent the standard error of the mean. Dots are connected between each type of pseudoword and its control.

We designed our pseudoword traps for two-by-two comparisons: orthographic traps versus words approximations, transpositions versus double substitutions, and mirror versus single substitutions. Figure 5 shows the data for these comparisons, which we consider in turn.

#### Impact of Orthographic Traps.

Our mixed effect analysis (RT: marginal r^2^: 0.020, conditional r^2^: 0.328; accuracy: marginal r^2^: 0.075, conditional r^2^: 0.285) only showed a significant main effect of pseudoword type on accuracy, *χ*^2^(1) = 32.35, *p* < .001, but not on RT, *F*(1, 147.36) = 0.038, *p* = 0.85, meaning that when they were correct, students were equally fast to classify orthographic traps and word approximations. We also found a significant effect of fluency quintile on both RT and accuracy (RT: *F*(4, 1428.30) = 37.10, *p* < .0001; accuracy: *χ*^2^(4) = 393.42, *p* < .001) and a significant interaction between fluency and pseudoword type only for RT (RT: *F*1(4, 19168.23) = 3.10, *p* = 0.015; accuracy: *χ*^2^(4) = 2.80, *p* = 0.59), which was preserved in z-transformed RTs, *F*(4, 20407.0) = 2.81, *p* = 0.024. Performing simple effect analysis, we found that this interaction was due to the fact that word approximations tended to be classified faster than orthographic traps, except in the least fluent students. We observed that OLD20, computed using Lexique 3.83 (New et al., 2004) as the reference lexicon, was lower for orthographic traps (mean = 2.09, range = 1.05–3.35) than for word approximations (mean = 2.20, range = 1.20–3.70). As this difference was significant, *F*(1, 30018) = 386.6, *p* < 0.001, we then examined whether the effects described above were still significant when orthographic similarity to real words was accounted for. Adding OLD20 as a covariate in our mixed-effect model, we found no significant difference in our results. The main effect of pseudoword type was still significant on accuracy, *χ*^2^(1) = 29.93, *p* < .001, but not on RT, *F*(1, 146.23) = 0.12, *p* = 0.73. The main effect of fluency quintile was still significant on both accuracy, *χ*^2^(4) = 393.38, *p* < .001, and RT, *F*(4, 1428.34) = 37.10, *p* < .001, and the interaction between fluency and pseudoword type was still significant on RT only (RT: *F*(4, 19168.15) = 3.11, *p* = 0.015; accuracy: *χ*^2^(4) = 2.77, *p* = 0.60).

#### Effect of Letter Transpositions.

The results of our mixed effect models (RT: marginal r^2^: 0.029, conditional r^2^: 0.261; accuracy: marginal r^2^: 0.075, conditional r^2^: 0.296) confirmed our expectations. For both RT and accuracy, we found a main effect of pseudoword type (RT: *F*(1, 175.21) = 11.60, *p* = 0.0008; accuracy: *χ*^2^(1) = 26.70, *p* < .001) meaning that transpositions were harder to classify than double substitutions. Initially, we found that the better the reader, the better the overall performance as highlighted by the main effect of fluency, (RT: *F*(4, 1403.38) = 46.75, *p* < .0001; accuracy: *χ*^2^(4) = 403.63, *p* < .001), in the absence of an interaction with pseudoword type (RT: *F*(4, 21294.44) = 2.02, *p* = 0.088; accuracy: *χ*^2^(4) = 3.53, *p* = 0.473). However, looking at *z*-scores, we did find a significant interaction between type and fluency level, *F*(4, 22646.1) = 2.46, *p* = 0.043, confirming that the difference observed between the two type of pseudowords is steeper as fluency level increases. Random selection of items led to a small but significant imbalance in OLD20 between the two pseudoword categories, *F*(1, 30018) = 4.76, *p* = 0.029, with OLD20 for transposition (mean = 2.24, range = 1.30–3.85) being a bit higher than OLD20 for double substitutions (mean = 2.22, range = 1.15–3.85). Accounting for orthographic similarity in the mixed effect model, however, did not change our findings. The main effect of pseudoword type was still significant for both RT, *F*(1, 174.10) = 11.67, *p* < .001, and accuracy, *χ*^2^(1) = 26.72, *p* < .001, as well as the main effect of fluency (RT: *F*(4, 1403.20) = 46.76, *p* < .001; accuracy: *χ*^2^(4) = 403.63, *p* < .001). The interaction between fluency and pseudoword type was still not significant (RT: *F*(4, 21294.25) = 2.02, *p* = 0.088; accuracy: *χ*^2^(4) = 3.53, *p* = 0.47).

#### Processing Mirror Substitutions.

Surprisingly, our results on this type of pseudoword departed from our prediction that mirror substitutions should be more difficult than single substitutions (figure 5). Our mixed effect model (RT: marginal r^2^: 0.031, conditional r^2^: 0.314; accuracy: marginal r^2^: 0.061, conditional r^2^: 0.282) showed a significant main effect of pseudoword type on both RT and accuracy (RT: *F*(1, 174.64) = 11.04, *p* = 0.0011; accuracy: *χ*^2^(1) = 13.73, *p* < .001), but with a difference in favor of mirror substitutions. There was a significant main effect of fluency quintile (RT: *F*(4, 1422.50) = 46.80, *p* < .0001; accuracy: *χ*^2^(4) = 383.65, *p* < .001) and a significant interaction on accuracy only (RT: *F*(4, 20941.65) = 0.20, *p* = 0.94 ; accuracy: *χ*^2^(4) = 22.02, *p* < .001). Within each fluency quintile, faster and more accurate performance with mirror letters reached significance for all but the least fluent students, *χ*^2^(1) = 3.23, *p* = 0.072 - the converse of our predictions. This was still the case after accounting for orthographic similarity (OLD20), which was higher for mirror substitutions (mean = 2.22, range = 1.30–3.85) than for single substitutions (mean = 2.08, range = 1.00–3.85), *F*(1, 30018) = 594.6, *p* < 0.001. The main effect of pseudoword type was still significant for both RT and accuracy (RT: *F*(1, 173.57) = 14.83, *p* < .001; accuracy: *χ*^2^(1) = 11.77, *p* < .001), as was the main effect of fluency quintile (RT: *F*(4, 1422.54) = 46.81, *p* < .001; accuracy: *χ*^2^(4) = 383.69, *p* < .001). The interaction between fluency and pseudoword type was still only significant on accuracy (RT: *F*(4, 20942.45) = 0.21, *p* = 0.93; accuracy: *χ*^2^(4) = 21.99, *p* < .001).

### Predicting Fluency Score Using Lexical Decision Results

Our last goal was to see whether the fluency score of each student could be predicted from his or her Lexical Decision results. To test this prediction, we extracted five LD parameters for each student: median RT; global error rate; slope of the length effect on RT for all the stimuli; slope of the frequency effect on RT for words; and efficiency score, defined as the ratio of accuracy by mean RT (note that this parameter, like fluency, evaluates the number of correct words per unit of time).

**Figure 6.**
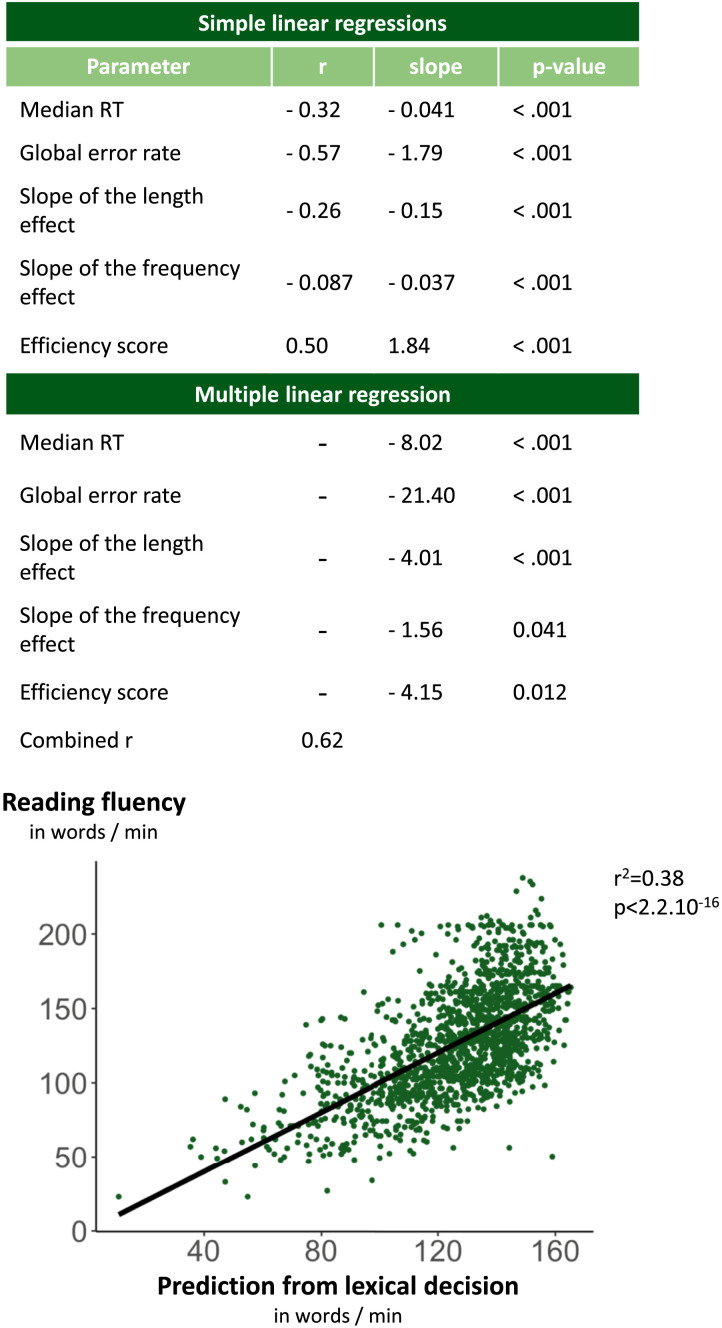
Predicting individual participants’ reading fluency from their results on the lexical decision task.

The results appear in Figure 6. Simple linear regressions indicated that all five parameters were predictive of text reading fluency, with LD global error rate being the most predictive one (r^2^ = 0.35). A multiple linear regression showed that all five parameters made significant and independent contributions, with the overall r^2^ reaching 0.38. Thus, we conclude that LD measures can predict about 40% of the variance in a one-minute text fluency reading test.

## DISCUSSION

Our primary goal was to evaluate the lexical decision performance of 6^th^ grade students at different levels of reading fluency, a known predictor of school reading comprehension (Fuchs et al., 2001; Hudson et al., 2005; National Reading Panel, 2000; Pinnell et al., 1995). Our results converge with prior research comparing LD performance across grades, children versus adults, or normal readers versus dyslexics, as further discussed below—but the originality of the present work is to thoroughly characterized the variability in reading fluency in a large sample of students within a single grade year (6^th^ grade). Our findings corroborate teachers’ reports of a large diversity of reading ability when students arrive in their first year of middle school. Based on student fluency scores, the best performing quintile had already reached adult reading levels, reading more than 200 words per minute, while three quarters of students in the worst quintile lagged behind 3^rd^ grade expectations (14.3% of our subsample), a striking result with practical implications. Note that this result cannot be attributed solely to the COVID crisis, as this percentage at the national scale remain unchanged over the following three years (2021: 16.0% / 2022: 15.2% / 2023: 16.8%) (Andreu et al., 2022, 2023a, 2023b). National statistics suggest that, depending on the definition of literacy, more than 10% of the French population is struggling with reading, and more than 5% remains functionally illiterate when they leave school and enter active life (Chabanon & Rosenwald, 2020). The present results suggest that the LD test in 6^th^ grade may pick such difficulties at a moment where they might still be acted upon.

Our findings are largely compatible with the hypothesis of two different pathways for reading words and pseudowords (Castles, 2006; Coltheart et al., 2001; Di Filippo et al., 2006). In line with previous research, we found a main effect of lexicality, with faster responses to known lexical items. The presence of a significant length effect on pseudoword RTs fits with the hypothesis that pseudowords are deciphered via a slow sublexical route. The decrease in the length effect as student’s fluency level increases provides an estimate of the effectiveness of their procedure: the better the students are at reading, the faster their lexical and sublexical pathways.

The impact of length on pseudoword reading stands in contrast with what was reported by Juphrad and colleagues who found no length effect on pseudoword RT in skilled readers (Juphard et al., 2004). The type of pseudowords used in the tasks can possibly explain the difference. Juphrad et al’s pseudowords were closest to our trigrams, most of which are orthographically distant from real words, while their words were of very high frequency (minimum 134 per million). Thus, it was far easier for their readers to base their decisions on lexicality, which may explain the absence of a length effect on pseudowords’ RT. In our study, however, the presence of rare words, as well as the frequent proximity of pseudowords to real words, may have forced participants to rely more heavily on decoding reading procedures.

A subtle aspect of our results is the interaction of length and lexicality on error rates only: while RTs systematically increased with length for both words and pseudowords, errors decreased slightly with length for words only. We suggest that this effect could potentially be linked to the lexical neighborhood density, which decreased for words, thus facilitating word identification, and partially counteracting the length effect. Indeed, as mentioned in the method, the OLD20 of our items was significantly correlated to length for both word frequencies and pseudoword types. As this could also be seen as a potential confound, we ran again all our models while adding OLD20 as a covariate. The length effect decreased but was still significant. This suggests a competition between these two effects but OLD20 may not be a perfect estimator of the lexical effect.

Access to the mental lexicon was evaluated by varying the frequency of the word stimuli. As expected, we found a significant frequency effect on both RT and accuracy. The absence of an interaction with fluency in the *z*-score analysis indicated that, frequency is a variable that primarily affects the decision stage and whose amplitude therefore covaries with the standard deviation of RTs. Once corrected for the overall greater slowness and variability of responses in the less fluent students, the frequency effect had the same size in all fluency quintiles, suggesting that for words they know, all students rely on a similar lexical procedure. This result is in line with those observed by Burani et al (Burani et al., 2002) in younger children, where they showed a frequency effect on naming latencies but no significant interaction between word frequency and grade.

Delving deeper, we found RT interactions between length and frequency to be dependent on fluency. All readers except the least fluent ones used lexical strategies to read very frequent words. The findings for our best readers regarding lexicality, length and frequency effects were consistent with those described by Araujo et al in Portuguese 3^rd^ to 5^th^ graders (Araújo et al., 2014). Our poorest readers, however, were similar to previously reported cases of dyslexic readers, as evidenced by a length effect on RT to words, thus betraying a strategy of accurate but sublexical reading even for frequent words (Araújo et al., 2014; Zoccolotti et al., 2005). In other words, our poorest readers managed to correctly judge a majority of frequent words but did so by first identifying them through a slow sublexical reading process.

The second goal of our study was to examine the impact of different types of pseudowords on response time and error rate. In general agreement with previous research, we found that stimuli that were most similar to real French words yielded the highest error rate (Bergmann & Wimmer, 2008; Grainger et al., 2012). Going further, we systematically compared orthographic traps with trigram-based approximations, transpositions with double substitutions, and mirror with single substitutions. We found greater mistakes on orthographic traps (homophones) than on trigram controls. Such errors are mainly due to regularizations of letter sounds, which is a marker of the use of the sublexical procedure. A possible interpretation of the reversed effect found on RT for least fluent students, with orthographic traps being classified faster than words approximation, could be that as we only looked at RTs to correct answers, and the least fluent students made a lot of errors in this category, they only classified correctly the most obvious items.

In line with prior research, our results revealed that transposed letters led to increased response times (RT) and error rates when compared to letter substitutions (Chambers, 1979; Grainger et al., 2012). Notably, this effect became more pronounced as we compared the least fluent readers to the most fluent ones. This observation can be interpreted as an indication of the growing reliance on the lexical procedure, where all letters are processed simultaneously. As a result, there is a higher likelihood of letter position confusions.

Finally, looking at mirror and single substitutions, we found a surprising effect: mirror substitutions were classified faster and more accurately than single substitutions. What could be the underlying reason of this surprising effect? A first possibility is that words with mirror letters are more likely to access the mental lexicon (e.g. ‘balance’ would be activated in response to ‘dalance’, more than to ‘falance’). Subsequently, top-down feedback from the lexicon might facilitate the detection of the erroneous letter ‘d’, while control pseudowords like ‘falance’ would not benefit from this lexical feedback. To probe the possible contribution of the lexical route, we tested for an effect of the frequency of the original word on these two categories of pseudowords. There was no main effect of frequency (RT: *F*(1, 173.64) = 0.20, *p* = 0.65; accuracy: *χ*^2^(1) = 0.12, *p* = 0.72) nor, crucially, any interaction of frequency with pseudoword type (RT: *F*(1, 172.55) = 0.08, *p* = 0.78; accuracy: *χ*^2^(1) = 0.67, *p* = 0.41). Thus, the lexical route does not appear to contribute much to the processing of those pseudowords, if at all.

A second possibility is that some mirror-letter substitutions led to stronger violation of the orthographic statistics of French, which could have facilitated the rejection of those pseudowords. Indeed, in French, the letter ‘q’ is very rare and is almost always followed by the letter ‘u’. However, when p was substituted with a q, this graphotactic rule was violated in all but one of our mirror-substituted pseudowords containing a ‘q’, leading to very implausible pseudowords (e.g. ‘qrochain’). To test this idea, we removed all items with a ‘q’ substitution (50% of the presented items) and repeated our mixed effect analysis. The main effect of pseudoword type vanished for both RT and accuracy (RT: *F*(1, 133.21) = 2.19, *p* = 0.14 ; accuracy: *χ*^2^(1) = 1.43, *p* = 0.231). There was also no interaction with fluency on RT, *F*(4, 15227.15) = 1.84, *p* = 0.12, and a minor one on accuracy: *χ*^2^(4) = 12.87, *p* = 0.012. This finding suggests that our paradoxical effect on RT (more efficient processing of mirror substitutions) was entirely due to the specifics of the letter ‘q’.

It remains to be explained why, once this spurious effect was removed, mirror letters still did not pose more difficulties than other letter substitutions for French middle-school readers. One possible interpretation could be that the students we tested have undergone five years of formal reading. Letter mirroring typically vanishes in the first few years of reading acquisition (Cornell, 1985), beyond which only rare dyslexic students are expected to exhibit the effect (McCloskey & Rapp, 2000). Although mirror-letter generalization remained detectable in adult readers using subliminal priming (see Duñabeitia et al., 2011; Lin & Ryan, 2007; Perea et al., 2011), the present findings indicate that it is not detectable in the present LD task with highly visible conscious targets. Further experiments should use minimal contrasts between supraliminal and subliminal conditions to investigate the time course and the conditions of emergence of a residual mirror effect as a function of reading fluency and conscious perception. Overall, with our models, we were able to explain between 25% and 33% of the variance in response time and accuracy.

Our final goal was to assess the correlation between LD and text reading fluency. We replicated, in adolescents, the prior finding that accuracy is a better predictor of oral reading ability than RT, a finding established in younger children, as well as in different languages (Gijsel et al., 2004; van Bon et al., 2004; Yeatman et al., 2021). Previous research has highlighted a correlation of r = 0.91 for LD and oral word reading (Yeatman et al., 2021). Here we achieved r = 0.62 by using multilinear regression combining median RT, global error rate, slope of the length effect, slope of the frequency effect and efficiency score (thus predicting 38% of the total variance in fluency scores). Our correlation was smaller than the one found by Yeatman et al., perhaps due to a shorter test or to a noisier, more distracting school environment used for measurement. However, the correlation is far from negligible, given that the small number of items seen by each subject and the fact that the least fluent students produced fewer correct responses on which to collect RTs.

**Figure 7.**
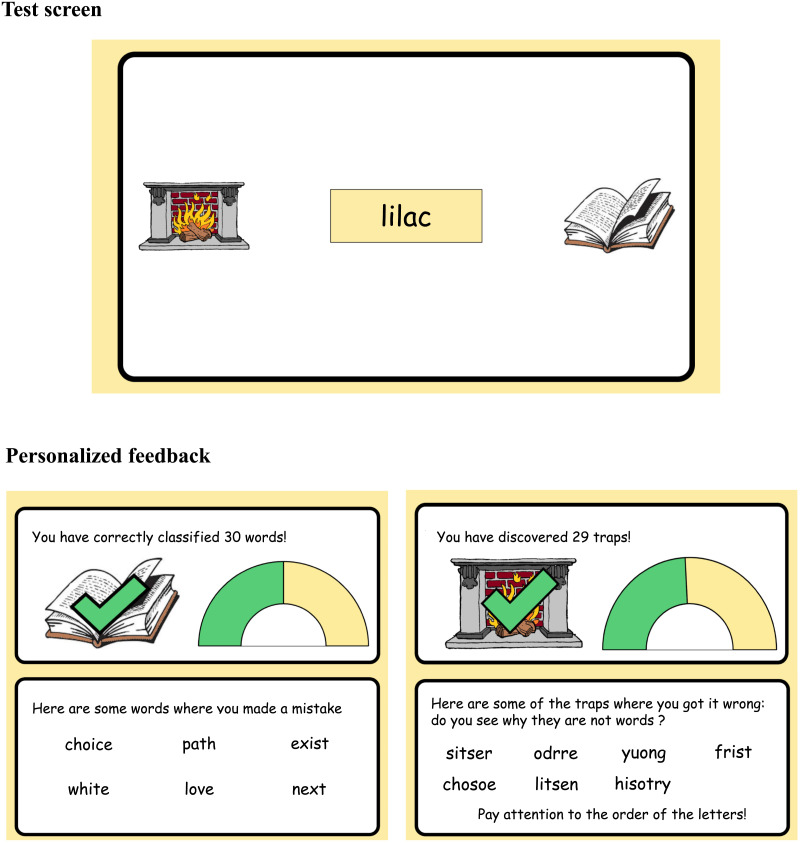
Gamified version of the lexical decision task. Students are asked to send each stimulus into the dictionary if it is a word, and into the fireplace if it is a trap. At the end of the session, they receive feedback on their performance, including the number of errors on words and pseudowords and examples of errors. For words, errors are sorted by their frequency. For pseudowords, only the stimuli from the category in which the student made the most errors are displayed. A tip on how to avoid these traps is given.

From a practical point of view, these results support the use of LD as a complementary test to oral reading fluency, one that can reveal details of the reading processes in students who show obvious difficulties on the fluency test (Balota et al., 2006; Seidenberg & McClelland, 1989). For example, our poorest readers showed a length effect on RTs to frequent words, suggesting that they know these words but identify them using a sublexical procedure, similarly to dyslexic students (Araújo et al., 2014; Castles, 2006). This insight from LD, which could not be obtained from fluency alone, may help flag students requiring special intervention. To this aim, we have begun to introduce teachers and students with a gamified version of our LD test (Figure 7). At the end of this version of the test, students and teachers are provided with a summary of the main results, broken down into two categories: first, the results on words, with examples of errors made in the most frequent category; and second, the results on pseudowords, with examples of the student’s main error type. A tip on how to avoid these types of errors in the future is added at the bottom of the page. In future research, we plan to test the usefulness of this gamified lexical decision test for both students and teachers.

There are several limitations to this work. As the Ministry of Education carried out the recruitment, we were not able to formally exclude dyslexic students from our participants. Thus, it is likely that part of the group of students with the greatest difficulties was in fact composed of dyslexics. Similarly, we were not able to check for the presence of other learning disabilities that might have disrupted the test, such as attention deficit disorder. We were also not able to test students twice or more in order to assess test-retest reliability, neither on the LD task nor on the fluency test (but their mutual correlation, r^2^ = 38%, provides a lower bound on their reliability). Furthermore, the great diversity of items presented to each child left little room for more than 2 or 3 repetitions of the same condition (2 repetitions of each length × type combination for pseudowords, 3 repetitions of each length × frequency combination for words). The high error rates on pseudowords therefore made it impossible to analyze the length effect within each type of pseudoword.

## CONCLUSIONS

In summary, our study showed that the variations in lexical decision results within a grade in middle school mimic those observed in cross-sectional studies, with a larger length effect for the least fluent students, but a similar frequency effect (once corrected for overall slowness). We suggest that LD cannot replace other oral reading tests such as fluency, but can be used to provide an additional assessment that sheds light on the efficiency of the two reading routes, i.e. lexical and sublexical reading procedures. LD has now proven its correlation with other tasks of oral reading. Given its ease of use, without requiring overt spoken responses or manual scoring, LD provides an excellent tool to differentiate between readers in need of extra practice, and those with more serious deficits marked by an inability to establish an efficient mental lexicon.

## ACKNOWLEDGMENTS

We gratefully acknowledge the Direction de l’Evaluation, de la prospective et de la performance (DEPP) of the French National Ministry for Education for its assistance in the deployment of the tests and the data collection and Johannes Ziegler, Mathias Sablé-Meyer and Aakash Agrawal for their advice on data analysis.

## FUNDING INFORMATION

This work was funded by INSERM, Collège de France, CEA, DEPP, the Collége de France foundation (C.S.) and the Clermont-Tonnerre foundation. M.L. was supported by a CIFRE grant from the CERENE schools and the French national agency ANRT.

## ETHICS STATEMENT

This project received ethics approval from the Comite d’Évaluation Éthique d’Établissement (C3E) of the Paris-Saclay University (10/11/2020, project reference: CER-Paris-Saclay-2020-068).

Parents gave their consent for the whole framework of the National Evaluations. Students gave their assent prior to their inclusion in the study.

## DATA AVAILABILITY STATEMENT

Raw data were collected by the French National Ministry for Education. Participants of this study did not give written consent for their data to be shared publicly, so supporting data is not available.

## Supplementary Material


