# Supplementary material for: Examining the Impact of Reading Fluency on Lexical Decision Results in French 6th Graders
Source: Open Mind (Camb). 2024 May 5;8:535–57. doi: 10.1162/opmi_a_00140 (PMC11093403; doi:10.1162/opmi_a_00140)

# Examining the Impact of Reading Fluency on Lexical Decision Results on French 6th graders

Supplementary materials

Marie Lubineau a,b,c*, Cassandra Potier Watkins a,b, Hervé Glasel c, Stanislas Dehaene a,b

a Cognitive Neuroimaging Unit, CEA DSV/I2BM, INSERM, Université Paris Sud, Université Paris-Saclay, NeuroSpin Center, Gif-sur-Yvette, France; b Collège de France, Paris, France ; c Reference centre for the neuropsychological evaluation of children (CERENE), Paris, France

Correspondence concerning this article should be addressed to Marie Lubineau, [marie.lubineau@college-de-france.fr](mailto:marie.lubineau@college-de-france.fr); <https://orcid.org/0000-0002-3084-5958>.

**Table S1**

*Results of the different models on response time with fluency as a continuous variable.*

| **Length*lexicality*fluency score** | | | | | | |
| --- | --- | --- | --- | --- | --- | --- |
|  | Num df | | Den df | F | p |  |
| Length | 1 | | 1061.6 | 69.87 | <.001 | *** |
| Lexicality | 1 | | 1062.1 | 209.22 | <.001 | *** |
| Fluency score | | 1 | 1491.0 | 221.19 | <.001 | *** |
| Length*lexicality | 1 | | 1061.7 | 7.59 | 0.006 | ** |
| Length*fluency score | 1 | | 135889.5 | 187.41 | <.001 | *** |
| Lexicality*fluency score | 1 | | 136068.9 | 51.84 | <.001 | *** |
| Length*lexicality*fluency score | 1 | | 135898.0 | 0.018 | 0.89 |  |
| **Word frequency*fluency score** | | | | | | |
|  | Num df | | Den df | F | p |  |
| Word frequency | 3 | | 557.26 | 33.44 | <.001 | *** |
| Fluency score | 1 | | 1483.56 | 204.54 | <.001 | *** |
| Word frequency*fluency score | 3 | | 70609.04 | 6.30 | <.001 | *** |
| **Word frequency*length*fluency score** | | | | | | |
|  | Num df | | Den df | F | p |  |
| Length | 1 | | 554.31 | 22.93 | <.001 | *** |
| Word frequency | 3 | | 552.14 | 34.48 | <.001 | *** |
| Fluency score | 1 | | 1483.35 | 204.68 | <.001 | *** |
| Length*word frequency | 3 | | 554.16 | 0.72 | 0.54 |  |
| Length*fluency score | 1 | | 70627.09 | 115.73 | <.001 | *** |
| Word frequency*fluency score | 3 | | 70607.56 | 6.28 | <.001 | *** |
| Length*word frequency*fluency score | 3 | | 70605.77 | 3.85 | 0.009 | ** |
| **Pseudoword type*fluency score – orthographic trap VS word approximation** | | | | | | |
|  | Num df | | Den df | F | p |  |
| Pseudoword type | 1 | | 147.21 | 0.062 | 0.80 |  |
| Fluency score | 1 | | 1453.75 | 145.27 | <.001 | *** |
| Pseudoword type*fluency score | 1 | | 19213.39 | 7.74 | 0.005 | ** |
| **Pseudoword type*fluency score – transposition VS double substitution** | | | | | | |
|  | Num df | | Den df | F | p |  |
| Pseudoword type | 1 | | 174.77 | 11.63 | <.001 | *** |
| Fluency score | 1 | | 1448.10 | 165.46 | <.001 | *** |
| Pseudoword type*fluency score | 1 | | 21365.72 | 0.066 | 0.80 |  |
| **Pseudoword type*fluency score – mirror substitution VS single substitution** | | | | | | |
|  | Num df | | Den df | F | p |  |
| Pseudoword type | 1 | | 174.35 | 11.13 | 0.001 | ** |
| Fluency score | 1 | | 1468.31 | 168.11 | <.001 | *** |
| Pseudoword type*fluency score | 1 | | 21007.90 | 1.53 | 0.22 |  |

**Table S2**

*Results of the different models on accuracy with fluency as a continuous variable.*

| **Length*lexicality*fluency score** | | | | | |
| --- | --- | --- | --- | --- | --- |
|  | df | | χ^2^ | p |  |
| Length | 1 | | 0.01 | 0.926 |  |
| Lexicality | 1 | | 92.73 | <.001 | *** |
| Fluency score | | 1 | 605.83 | <.001 | *** |
| Length*lexicality | 1 | | 14.50 | <.001 | *** |
| Length*fluency score | 1 | | 5.24 | 0.022 | * |
| Lexicality*fluency score | 1 | | 10.93 | <.001 | *** |
| Length*lexicality*fluency score | 1 | | 2.09 | 0.148 |  |
| **Word frequency*fluency score** | | | | | |
|  | df | | χ^2^ | p |  |
| Word frequency | 3 | | 231.52 | <.001 | *** |
| Fluency score | 1 | | 448.61 | <.001 | *** |
| Word frequency*fluency score | 3 | | 23.95 | <.001 | *** |
| **Word frequency*length*fluency score** | | | | | |
|  | df | | χ^2^ | p |  |
| Length | 1 | | 9.99 | .002 | ** |
| Word frequency | 3 | | 235.24 | <.001 | *** |
| Fluency score | 1 | | 451.70 | <.001 | *** |
| Length*word frequency | 3 | | 3.66 | 0.301 |  |
| Length*fluency score | 1 | | 6.40 | 0.011 | * |
| Word frequency*fluency score | 3 | | 24.42 | <.001 | *** |
| Length*word frequency*fluency score | 3 | | 7.89 | 0.050 |  |
| **Pseudoword type*fluency score – orthographic trap VS word approximation** | | | | | |
|  | df | | χ^2^ | p |  |
| Pseudoword type | 1 | | 32.74 | <.001 | *** |
| Fluency score | 1 | | 415.76 | <.001 | *** |
| Pseudoword type*fluency score | 1 | | 0.35 | 0.552 |  |
| **Pseudoword type*fluency score – transposition VS double substitution** | | | | | |
|  | df | | χ^2^ | p |  |
| Pseudoword type | 1 | | 27.07 | <.001 | *** |
| Fluency score | 1 | | 396.79 | <.001 | *** |
| Pseudoword type*fluency score | 1 | | 0.00 | 0.971 |  |
| **Pseudoword type*fluency score – mirror substitution VS single substitution** | | | | | |
|  | df | | χ^2^ | p |  |
| Pseudoword type | 1 | | 13.64 | <.001 | *** |
| Fluency score | 1 | | 371.35 | <.001 | *** |
| Pseudoword type*fluency score | 1 | | 10.07 | .002 | ** |

**Figure S1**

*Variation of OLD 20 with length for our different types of stimuli*


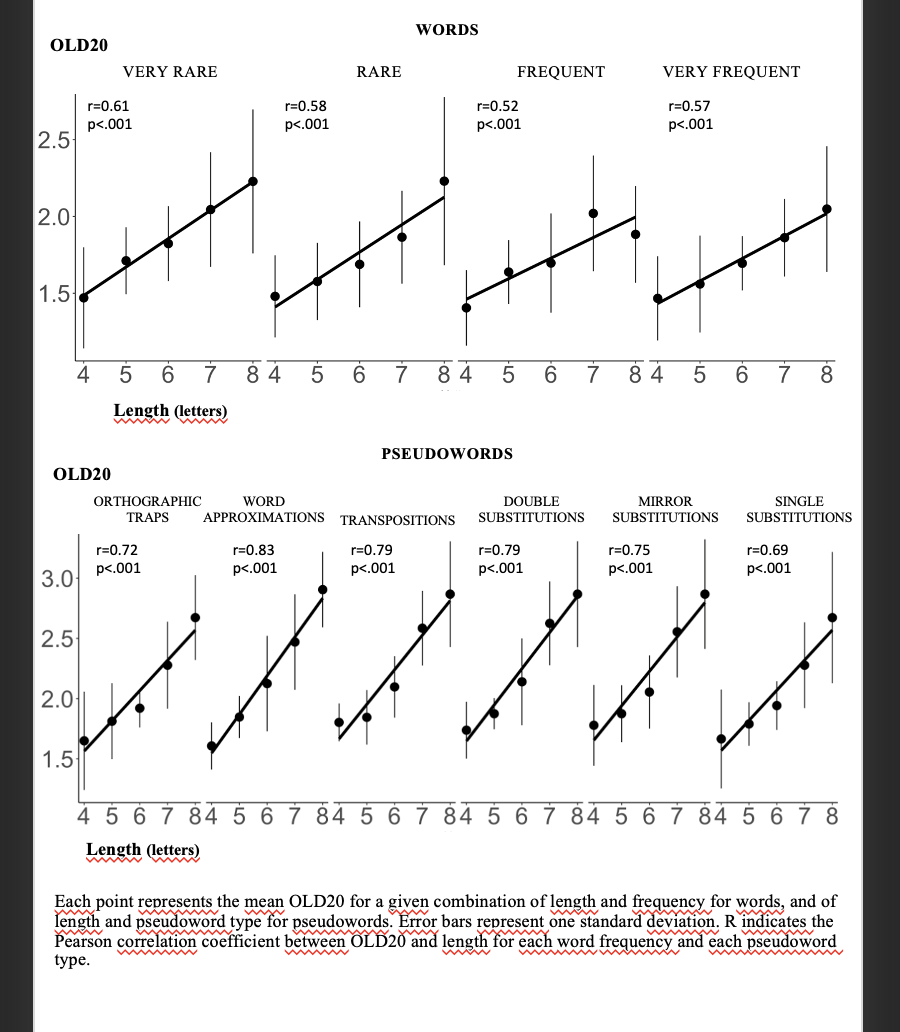

Supplement: Supplementary file 1 [file opmi-08-535-s001.docx]
